# Proteomic Interaction Patterns between Human Cyclins, the Cyclin-Dependent Kinase Ortholog pUL97 and Additional Cytomegalovirus Proteins

**DOI:** 10.3390/v8080219

**Published:** 2016-08-18

**Authors:** Mirjam Steingruber, Alexandra Kraut, Eileen Socher, Heinrich Sticht, Anna Reichel, Thomas Stamminger, Bushra Amin, Yohann Couté, Corina Hutterer, Manfred Marschall

**Affiliations:** 1 Institute for Clinical and Molecular Virology, Friedrich-Alexander University of Erlangen-Nürnberg, 91054 Erlangen, Germany; mirjam.steingruber@viro.med.uni-erlangen.de (M.S.); anna.reichel@viro.med.uni-erlangen.de (A.R.); thomas.stamminger@viro.med.uni-erlangen.de (T.S.); corina.hutterer@viro.med.uni-erlangen.de (C.H.); 2 Laboratoire Biologie à Grande Echelle (BGE), Exploring the Dynamics of Proteomes (EDyP), Université Grenoble Alpes, iRTSV-BGE, 38000 Grenoble, France; alexandra.kraut@cea.fr (A.K.); yohann.coute@cea.fr (Y.C.); 3 CEA, iRTSV-BGE, Grenoble 38000, France; 4 INSERM, BGE, Grenoble 38000, France; 5 Division of Bioinformatics, Institute of Biochemistry, Friedrich-Alexander University of Erlangen-Nürnberg, 91054 Erlangen, Germany; eileen.socher@fau.de (E.S.); heinrich.sticht@fau.de (H.S.); 6 Division of Biochemistry, Department of Biology, Friedrich-Alexander University of Erlangen-Nürnberg, 91058 Erlangen, Germany; bushra.amin@fau.de

**Keywords:** human cytomegalovirus, CDK ortholog pUL97, human cyclins, cyclin-associated protein complexes, proteomic analysis, molecular modeling

## Abstract

The human cytomegalovirus (HCMV)-encoded cyclin-dependent kinase (CDK) ortholog pUL97 associates with human cyclin B1 and other types of cyclins. Here, the question was addressed whether cyclin interaction of pUL97 and additional viral proteins is detectable by mass spectrometry-based approaches. Proteomic data were validated by coimmunoprecipitation (CoIP), Western blot, in vitro kinase and bioinformatic analyses. Our findings suggest that: (i) pUL97 shows differential affinities to human cyclins; (ii) pUL97 inhibitor maribavir (MBV) disrupts the interaction with cyclin B1, but not with other cyclin types; (iii) cyclin H is identified as a new high-affinity interactor of pUL97 in HCMV-infected cells; (iv) even more viral phosphoproteins, including all known substrates of pUL97, are detectable in the cyclin-associated complexes; and (v) a first functional validation of pUL97-cyclin B1 interaction, analyzed by in vitro kinase assay, points to a cyclin-mediated modulation of pUL97 substrate preference. In addition, our bioinformatic analyses suggest individual, cyclin-specific binding interfaces for pUL97-cyclin interaction, which could explain the different strengths of interactions and the selective inhibitory effect of MBV on pUL97-cyclin B1 interaction. Combined, the detection of cyclin-associated proteins in HCMV-infected cells suggests a complex pattern of substrate phosphorylation and a role of cyclins in the fine-modulation of pUL97 activities.

## 1. Introduction

The human cytomegalovirus (HCMV) is a pathogen with worldwide distribution [1]. While primary infection in immunocompetent individuals mostly remains asymptomatic, HCMV can cause a common opportunistic infection in immunocompromised individuals, including transplant recipients, tumor and AIDS patients. These complex clinical situations are frequently associated with end-organ disease, graft rejection or cardiovascular symptoms [2,3]. Notably, HCMV is the leading cause of congenital infection and fetal malformation in developed countries and has also been associated with excess mortality in the general population [3,4,5]. The currently licensed anti-HCMV drugs comprise the nucleoside analogue ganciclovir (GCV), its prodrug valganciclovir (VGCV), foscarnet (FOS) and cidofovir (CDV), all of which inhibit viral DNA synthesis [6]. However, antiviral therapy with these compounds often results in drug-resistant virus variants during long-term treatment and causes severe side effects such as myelo- and nephrotoxicity [7], so that there is a continued need for improved antivirals [8].

Protein kinases have moved into focus as potential novel targets of antiherpesviral drugs [9,10,11,12]. On the one hand, cyclin-dependent kinases (CDKs), which play functionally relevant roles during HCMV infection, are promising candidates. CDK inhibitors, such as roscovitine (targeting CDK1, 2, 5, 9), R25 (alsterpaullone; CDK1, 2, 5), R22 (CDK9) and LDC4297 (CDK7), have shown strong antiviral activities in cell culture models including both laboratory and clinically relevant virus strains [12,13,14,15,16]. On the other hand, herpesviral protein kinases, such as HCMV pUL97, may also represent attractive targets. During the last few years, a phase III study on anti-HCMV efficacy has been performed with the pUL97-targeting benzimidazole-type inhibitor maribavir (MBV); however, it did not reach primary end-points [17], so that very recently, a resumption of clinical investigations had to be restarted at the level of name patient programs and earlier clinical phases [18]. Other types of pUL97 inhibitors belonging to distinct chemical classes, such as Gö6976 and NGIC-I (indolocarbazoles), gefitinib, Ax7396, Vi7392 and Vi7453 (quinazolines), also exerted strong antiviral activity both in vitro and in vivo [19,20,21,22,23]. Although pUL97 is not absolutely essential for viral replication, the pharmacological inhibition of pUL97 results in a drastic reduction of viral replication efficiency [21,24,25].

As a multifunctional kinase, pUL97 phosphorylates several viral and cellular substrates and thus regulates important steps during HCMV replication, including gene expression, DNA replication, nuclear capsid egress, virion morphogenesis and cell cycle modulation [12,25]. Importantly, pUL97 represents a viral CDK ortholog due to CDK-related structural and functional characteristics. Sequence analysis and three-dimensional modeling of pUL97 showed structural similarity to CDK2 in the catalytic center and in functionally important residues of the ATP binding site [26]. Functional similarity to CDKs has been demonstrated by various experimental settings, such as a yeast complementation assay in which recombinantly expressed pUL97 rescued a *Saccharomyces cerevisiae* mutant lacking CDK activity [27]. In line with that, pUL97 and CDKs share identical substrates, such as nuclear lamins A/C, the retinoblastoma protein Rb, RNA polymerase II, translational elongation factor EF-1δ and histones, as well as the viral mRNA transporter pUL69 [13,26,27,28,29,30,31,32,33,34,35,36]. Notably, Rb is phosphorylated by CDKs and pUL97 at identical residues [15,27,34,37,38]. In addition, simultaneous experimental suppression of CDK and pUL97 activities increased the antiviral effect of MBV, pointing to a partially overlapping function between pUL97 and CDKs [39]. Thus, CDKs, as controllers of cell cycle progression, transcription, differentiation, apoptosis and neuronal functions [40,41], also play an important role during HCMV replication, acting on various levels of regulation. CDKs themselves are regulated by cyclin binding and phosphorylation [42]. Specifically, cyclins are known to confer substrate specificity on CDK-cyclin complexes, either via contributing to the affinity of substrate binding or via targeting CDKs to specific subcellular compartments [43,44]. During HCMV infection, cells show increased levels and activation of CDK-cyclin complexes (CDK1-cyclin B1, CDK2-cyclin E, CDK7-cyclin H, and CDK9-cyclin T1), as well as increased phosphorylated Rb and p53. In contrast, other subsets of CDK-cyclin complexes are down-modulated (CDK4-cyclin D, CDK6-cyclin D, and CDK2-cyclin A), consequently leading to an early S-phase arrest, termed pseudomitosis, offering favorable conditions for viral replication [12,39,45,46,47,48].

In the present study, we used high resolution mass spectrometry-based proteomics to investigate the molecular basis of the pUL97-cyclin interaction, emphasizing the functional relation between CDKs and the viral CDK ortholog pUL97. Differential modes of interaction of pUL97 with individual types of cyclins were detected in the proteomic settings and were supported by biochemical and bioinformatic analyses. Interestingly, the detection of viral phosphoproteins physically associated with cyclin coimmunoprecipitates strongly strengthens the hypothesis of a functional context that may promote the association of multimeric pUL97-cyclin-substrate complexes, possibly triggering selective phosphorylation. In particular, our proteomics-based data support earlier findings on the association of viral pUL97 with certain types of human cyclins, here specified as types B1, H and T1, thus leading to a refined concept of cyclin-mediated HCMV-host interaction.

## 2. Materials and Methods

### 2.1. Cell Culture, HCMV Infection and Transient Transfection

Human embryonic epithelial 293T cells (ATCC CRL-3216) were cultivated in Dulbecco’s modified Eagle’s medium (DMEM) containing 10% fetal calf serum (FCS) and primary human foreskin fibroblasts (HFFs) in minimum essential medium (MEM) containing 7.5% FCS. HCMV infection experiments were performed at a multiplicity of infection (MOI) of approximately 1.0 using HCMV strains AD169-GFP [49] and TB40 (stocks of both strains were grown on HFFs). Transfection of 293T cells with the expression plasmid pcDNA-UL97-Flag was performed using polyethyleneimine reagent (Sigma-Aldrich, Taufkirchen, Germany) as previously described [50].

### 2.2. Polyclonal Antisera and Monoclonal Antibodies

The following polyclonal (pAb) and monoclonal (mAb) antibodies were used: mAb-UL97 (kindly provided by T. Lenac and S. Jonjic; Department of Histology & Embryology, University of Rijeka, Croatia), pAb-UL97 (06-09, kindly provided by D.M. Coen, Harvard Medical School, Boston, MA, USA), mAb-cyclin B1 (sc-7393, Santa Cruz Biotechnology, Dallas, TX, USA; GNS11, Thermo Fisher Scientific, Waltham, MA, USA), pAb-cyclin B1 (sc-752, Santa Cruz Biotechnology), mAb-cyclin T1 (sc-271348, Santa Cruz Biotechnology), pAb-cyclin T1 (sc-10750, Santa Cruz Biotechnology), pAb-cyclin H (sc-609, Santa Cruz Biotechnology), pAb-cyclin A (sc-596, Santa Cruz Biotechnology), pAb-cyclin E (sc-198, Santa Cruz Biotechnology), pAb-cyclin D1 (sc-753, Santa Cruz Biotechnology), pAb-cyclin B2 (sc-22776, Santa Cruz Biotechnology), pAb-Fc (rabbit Fc fragment, Jackson ImmunoResearch Laboratories, West Grove, PA, USA), mAb-Flag (M2, Sigma-Aldrich, Taufkirchen, Germany), pAb-Flag (F7425, Sigma Aldrich), mAb–RNAP II (8WG16, BioLegend, San Diego, CA, USA), mAb-p-S5–RNAP II (AK4H8,BioLegend ), mAb-p-S7–RNAP II (4E12, Chromotek, Planegg-Martinsried, Germany), pAb-CDK2 (M2, Santa Cruz Biotechnology), mAb-Cyclin H (D-10, Santa Cruz Biotechnology), mAb-Cyclin E (E-4, Santa Cruz Biotechnology), pAb683 p-S807/811-Rb (Cell Signaling, Beverly, MA, USA), mAb-Rb (4H1, Cell Signaling), pAb-CDK7 (N-19, sc-56284, Santa Cruz Biotechnology), mAb-IE1p72 (63-27, kindly provided by W. Britt, University of Alabama, Birmingham, AL, USA), mAb-UL44 (BS510, kindly provided by B. Plachter, University of Mainz, Germany), and mAb-β-actin (AC-15, Sigma-Aldrich).

### 2.3. Coimmunoprecipitation (CoIP)

The 293T cells were seeded in 10 cm dishes at a cell number of 5 × 10^6^ and were transfected with expression plasmids coding for pUL97-Flag using the polyethylenimine transfection technique [50]. For mass spectrometry analysis, three 10 cm dishes per sample were used. Red fluorescent protein (RFP) was encoded by the pDsRed1-N1 reporter plasmid (BD Clontech, Palo Alto, CA, USA), and served as a transfection control. HCMV AD169-infected HFFs were cultivated in cell culture flasks (two per sample) and harvested at four to six days post-infection as indicated. Optionally, inhibitors were added to the culture media at indicated timepoints before harvesting the cells. Plasmid-transfected 293T cells and HCMV-infected HFFs were lysed in 500 µL CoIP buffer (50 mM Tris/HCl [pH 8.0], 150 mM NaCl, 5 mM EDTA, 0.5% NP-40, 1 mM phenylmethylsulfonyl fluoride (PMSF), 2 µg·mL^−1^ aprotinin, 2 µg·mL^−1^ leupeptin and 2 µg·mL^−1^ pepstatin) for 10 or 30 min on ice, respectively; centrifuged (14,000 rpm, 4 °C, 10–30 min) and incubated for about 3 h at 4 °C under rotation with antibody-coated Dynabeads^®^ Protein A (30 µL per sample; Thermo Fisher Scientific, Waltham, MA, USA). The precipitates were washed four times with CoIP buffer (1 mL each) before dividing the samples during the last washing step into aliquots to be used for quality control stainings and mass spectrometry analysis.

### 2.4. Quality Control of Purified Samples

To ensure high quality of the immunoprecipitated protein complexes, samples were analyzed both qualitatively and quantitatively. First, samples were analyzed to ensure successful CoIP of cyclins and pUL97 via standard Western blot (Wb) detection using antibodies as indicated (ECL staining, New England Biolabs, Frankfurt, Germany). Second, proteins were separated on 12.5% SDS-PAGE gels and Coomassie stained with InstantBlue^TM^ (Expedeon, San Diego, CA, USA) for one hour at room temperature under continuous gentle agitation. Thereafter, gels were subjected to silver staining procedures as previously described with a final staining step for up to 15 min [51].

### 2.5. Lentiviral Transduction and Selection of Stably Transduced HFFs

HFFs were subjected to lentiviral transduction and selected for stable expression of pUL97. To this end, replication-deficient lentiviruses were prepared by cotransfection of 293T cells with a pLenti6/V5-D-TOPO vector coding for pUL97 together with a packing plasmid mix coding for HIV-1 Gag/Pol, HIV-1 Rev and the envelope protein G of vesicular stomatitis virus (expression vectors pLP1, pLP2, and pVSV-G, respectively) using Lipofectamine 2000 (Invitrogen, Karlsruhe, Germany). Viral supernatants were harvested 48 h post-transfection, subjected to centrifugation to remove debris, filtered, and stored in aliquots at −80 °C. HFFs of a low passage number were incubated for 24 h with various amounts of lentiviral supernatant in the presence of 7.5 μg·mL^−1^ polybrene (Sigma-Aldrich, Taufkirchen, Germany). Next, virus supernatants were replaced by fresh media and cells were incubated for another 24 h before blasticidin (2 μg·mL^−1^, PAA Laboratories, Cölbe, Germany) was added in order to select for stably transduced cell populations.

### 2.6. Proteomic Analyses

Protein preparation and in-gel digestion were performed as described before [51]. In brief, immunoprecipitated complexes were solubilized in Laemmli buffer, stacked on top of a 4%–12% NuPAGE gel (Invitrogen, Karlsruhe, Germany) and stained by R-250 Coomassie Blue (BioRad, Hercules, CA, USA). Gel bands were then excised and proteins were in-gel digested using trypsin (Promega, Mannheim, Germany). Resulting peptides were analyzed by nanoliquid chromatography coupled to tandem mass spectrometry (Ultimate 3000 coupled to LTQ-Orbitrap Velos Pro, Thermo Scientific, Waltham, MA, USA) using a 120-min gradient. Peptides and proteins were identified through concomitant searches against Uniprot (*Homo sapiens* and human cytomegalovirus strain AD169 taxonomies), classical contaminants database (in-house generated) and the corresponding reversed databases using Mascot (version 2.5.1). The entirety of primary raw data is provided by Tables S1–S3. Proline software was used to filter the results (conservation of rank 1 peptides, peptide identification false discovery rate (FDR) < 1% as calculated based on peptide scores by employing the reverse database strategy, minimum peptide score of 25 and peptide length ≥ 7) before compilation, grouping and comparison of the protein groups from the different samples. To be considered as a potential binding partner of a bait, a protein had to exhibit a minimum weighted spectral count (WSC) of 3 (calculated as described in [52]) and be found at least 3-fold enriched compared to negative control samples (i.e., a minimum pseudocount of 1 was set in cases where no spectral count was detected in negative controls).

### 2.7. In Vitro Kinase Assay (IVKA)

The in vitro activity of HCMV kinase pUL97 was determined by IVKA analysis as described before [20,28,32]. pUL97-Flag and the catalytically inactive mutant pUL97(K355M)-Flag [21] were transiently expressed in transfected 293T cells and immunopurified by IP using mAb-Flag under conditions of increased stringency (500 mM NaCl) to prevent cyclin association. Recombinantly produced human cyclin B1 (2 µg; ab128445, Abcam, Cambridge, UK) and a mix of human histones 1–4 (20 µg; 10 223 565 001, Roche, Mannheim, Germany) were exogenously added to the reactions (2.5 µCi of [γ-^33^P] ATP). Samples were subjected to 12.5% SDS-PAGE separation, followed by Western blotting and autoradiography of the Wb membranes as exposed to BAS-2000 phosphorimager (Fuji Film. Co., Tokyo, Japan). The identity of radioactive products was verified by immuno-restaining of the Wb membranes.

### 2.8. Molecular Modeling

The homology model of pUL97 was prepared using human CDK2 (PDB code: 2JGZ; [53]) as a template (for details see [50]). The loop formed by the non-conserved amino acids 506-541 of pUL97 has not been modeled yet. The position and orientation of cyclin B1 and cyclin T1 relative to pUL97 was derived from the complex crystal structures of CDK2-cyclin B1 (PDB code: 2JGZ) and CDK9-cyclin T1 (PDB code: 3MI9; [54]), respectively. However, only the structure of unbound cyclin H (PDB code: 1KXU; [55]), but not that of a CDK-cyclin H complex, is presently available. Since cyclin H exhibits a higher structural similarity to cyclin T1 than to cyclin B1, the pUL97-cyclin H complex was modeled using the CDK9-cyclin T1 complex geometry as a template. All pUL97-cyclin complex structures were subsequently refined by an energy minimization with Amber14 [56]. pUL97-cyclin complex visualization was done with VMD 1.9.2 [57].

## 3. Results

### 3.1. Proteomic Detection of Protein Complexes Based on the Interaction between Cyclin B1 and Viral Kinase pUL97

**Figure 1:**
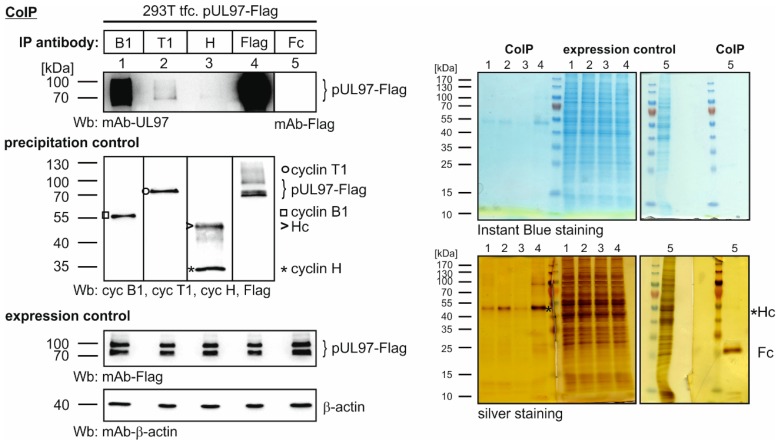
Coimmunoprecipitation of pUL97 with cyclins B1, T1 and H using protein lysates from plasmid-transfected (tfc. 293T cells). The 293T cells expressing pUL97-Flag were lysed two days post-transfection and endogenous cyclins were immunoprecipitated using polyclonal antibodies (pAbs). Immunoprecipitation of Flag-tagged pUL97 served as a positive control and the Fc fragment of rabbit antibodies was used as a background control (Hc, immunoglobulin heavy chain). Levels of expression and coimmunoprecipitation were monitored by parallel Western blot (Wb) analysis. Additional Instant Blue and silver stainings were applied to verify the quality of all protein samples.

As an initial step, the question was addressed whether an interaction of individual types of human cyclins and cytomegaloviral proteins might be detected using high-sensitivity methods. Recently, we described the interaction between the HCMV regulatory protein pUL69 [13,14,36] as well as HCMV kinase pUL97 and human cyclins [58]. The latter phenomenon was characterized by an especially strong pUL97-cyclin B1 interaction [50]. In these previous studies, the parameters of pUL97-cyclin interaction were determined by the use of well-established methods including coimmunoprecipitation, yeast two-hybrid and confocal colocalization approaches. In the present study, we analyzed the interaction between pUL97 and cyclins B1, T1 and H by applying coimmunoprecipitation-mass spectrometry-based proteomics. For this purpose, total lysates of transiently transfected, pUL97-expressing 293T cells were subjected to CoIP analysis using polyclonal antibodies directed against endogenous cyclins. Before performing mass spectrometry analysis on the samples, the reliability of expression levels as well as successful CoIP of pUL97 and cyclins in the absence of protein degradation was monitored by Wb, Instant Blue and silver stainings (Figure 1). Here, pUL97 showed very strong CoIP/Wb signals for cyclin B1 interaction, but much weaker signals for cyclin T1 and cyclin H (Figure 1, lanes 1–3). As an internal control, pUL97 was directly immunoprecipitated (Figure 1, lane 4; note that the very strong signal is additionally explained by the known capacity of pUL97 to oligomerize [59]). As an important novel point of our study, the data obtained by mass spectrometry coincided with the CoIP results, in that pUL97 was detected in complex with cyclins B1, T1 and H at levels with WSC ratios of 27, 4 and 5, respectively (Table 1; Fc fragment alone was used as a background control). For all three cyclins analyzed, the corresponding CDKs could be detected in the expected samples confirming the reliability of our setting. As a major finding, pUL97 association with the three types of human cyclins was most pronounced for cyclin B1 under these conditions of pUL97 overexpression in the absence of other viral proteins. This strong cyclin B1 interaction was also confirmed by the fact that no other cyclins were detected in the pUL97-specific coimmunoprecipitate (WSC 5; Table 1). However, as illustrated by the below-mentioned experiments using lysates from HCMV-infected cells, it should be stressed that pUL97-cyclin interaction may include more types of cyclins and that the selectivity of interaction may be modulated during the course of viral replication.

### 3.2. In HCMV-Infected Cells, but Not in Ectopically pUL97-Expressing Cells, Cyclin H is Abundant in the pUL97 Interactome Compared to Other Types of Cyclins

**Figure 2:**
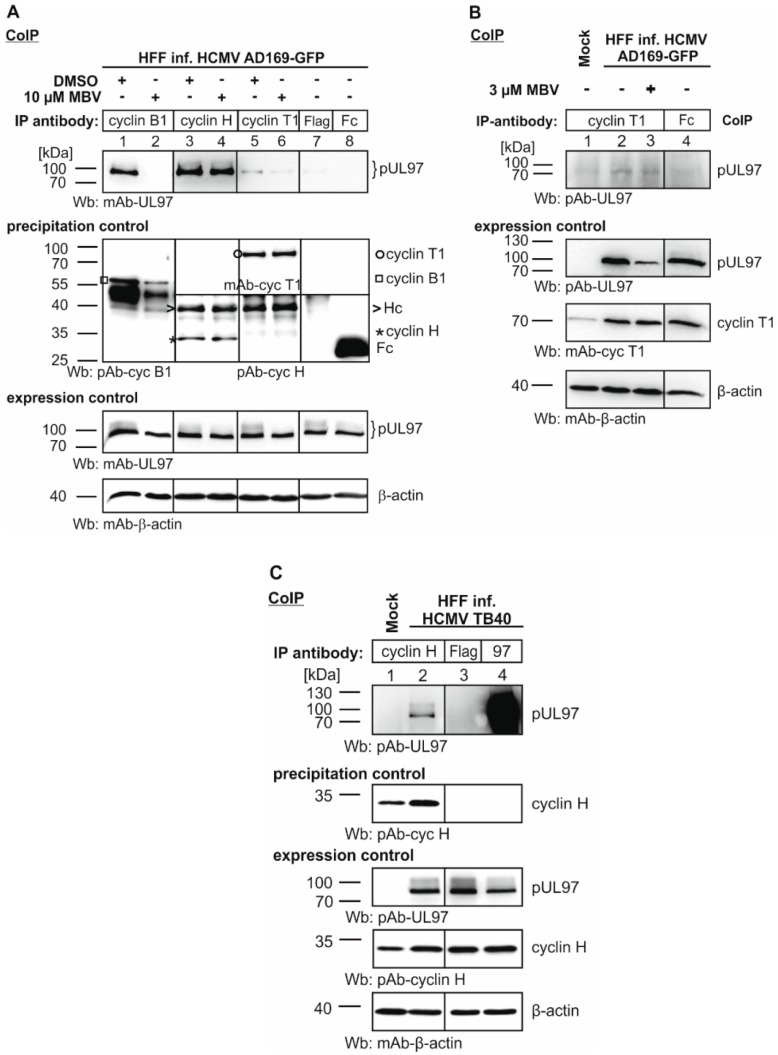
Coimmunoprecipitation of pUL97 with cyclins B1, T1 and H using protein lysates from HCMV-infected cells. (**A**) HFFs were infected with HCMV AD169-GFP for five days before 10 µM of pUL97 inhibitor maribavir (MBV) was added to the medium one hour prior to cell harvest. After lysis, endogenous cyclins B1, T1 and H were immunoprecipitated using polyclonal antibodies (panel CoIP). Anti-Flag antibody and a rabbit Fc antibody fragment were used as specificity controls. Levels of expression and CoIP were monitored in parallel by Wb analysis; (**B**) An additional CoIP experiment was performed (harvest of cells four days post-infection) to substantiate the specificity of pUL97-cyclin T1 interaction; (**C**) HCMV strain TB40 was used to address the question of strain independence of pUL97-cyclin H interaction (CoIP conditions as in (**A**).

In the next step, we used cyclin immunoprecipitates from total lysates of HCMV-infected fibroblasts. The CoIP samples were first analyzed by SDS-PAGE/Wb to obtain information about the presence and stability of all proteins of interest (Figure 2). In the Wb detection, a successful immunoprecipitation of the three analyzed cyclins and a strong expression of pUL97 in the protein lysates from HCMV-infected cells was demonstrated (precipitation and expression controls, respectively; upper panels). Of note, pUL97 was abundantly coimmunoprecipitated with cyclins H and B1 (Figure 2A, lanes 1 and 3), whereas cyclin T1 showed a much lower, but still specifically detectable level of pUL97 interaction (lanes 5–6, compared to specificity controls in lanes 7–8). The latter finding is consistent with earlier observations comparing cyclins B1 with T1 [50]. Using a densitometric quantitation of Wb signals from HCMV-infected HFF samples (with mean values derived from four independent experiments harvested at four, five or six days post-infection), the high-affinity interaction of cyclin H with pUL97 was illustrated (Table 2; 160% signal intensity for cyclin H compared to 100% for cyclin B1, taken as the reference, and a reduced level of 77% for cyclin T1). Interestingly, a completely different picture was obtained when pUL97 was ectopically expressed by transduction of HFFs (i.e., stably expressing HFFs selected after retroviral gene transfer). In this case, cyclin B1 interaction was most pronounced (Table 2; 100%), whereas cyclin H interaction was reduced to background levels (6%, compared to the Fc negative control of 4%) and cyclin T1 showed an intermediate level of interaction (42%). This apparently differential binding to pUL97 of the three cyclins was even more pronounced when proteins from transiently pUL97-transfected 293T cells were used as described above (3.1). Here, pUL97-cyclin B1 interaction was highly abundant (100%) compared to cyclin H (4%) and cyclin T1 (9%). This finding confirms and extends our earlier statement that pUL97 preferentially interacts with cyclin B1 in pUL97-overexpressing cells [50], but that pUL97 interacts with more types of cyclins in HCMV-infected cells. It should be noted, however, that even though presenting relative values, the varying efficiencies of CoIP antibodies may have an impact on signal intensities, so that our approach based on CoIP, Wb and mass spectrometry may be considered semi-quantitative. Yet, the novel data described in Figure 2 and Table 2 and Table 3 point to a probable modulation of cyclin interaction properties during HCMV replication. In particular, cyclin H appears to be subject to a major modulation, since it is incapable of interacting with pUL97 in ectopically expressing, transfected cells, but shows a strong pUL97 interaction in HCMV-infected cells.

Moreover, a second strain of HCMV, TB40, was used to substantiate our novel finding of cyclin H interaction with pUL97 in HCMV-infected fibroblasts. CoIP analysis demonstrated a specific signal of interaction (Figure 2C) and no difference was noted between the two strains AD169 and TB40. This finding is consistent with our earlier observations, which also included the wild-type-like strain Merlin, stating that pUL97-cyclin interaction, similar to pUL69-cyclin interaction, appears to be independent from viral strain and host cell type [13,36,56].

### 3.3. Complex Patterns of Interaction Are Detectable for Human Cyclins and the Associated Viral Kinase pUL97 as Well as Additional Viral Proteins

This point was strongly supported and further illustrated by mass spectrometry data. When using samples under the identical HCMV infection conditions in HFFs (four days post-infections), one of the strongest levels of pUL97 interaction was found for cyclin H (Table 3; relative WSC of 13) compared to other types of human cyclins, namely cyclins A, B1, E or T1 (WSC 5, b.c., 3 or 3, respectively), while cyclins B2 and D1 were negative. Interestingly, pUL97-cyclin B1 remained below cut-off in this measurement, whereas it was positive with WSC 6 and 13 in two independent measurements of another experiment (Table 4 and data not shown, respectively), so that the detectability of this complex by mass spectrometry appears to vary more than for other pUL97-cyclin complexes (including some possible variation of antibody efficiencies). All proteomic results are presented as ratios of WSCs between cyclin CoIP and Flag antibody CoIP which was used as a reference control for CoIP specificity (CoIP with a non-reactive Fc fragment was used as an additional negative control; Table 3, right lane Fc). For comparison to cyclin CoIPs, an anti-UL97 antibody was used for CoIP to confirm the reliability of the system (Table 3, lane pUL97; note that most of the pUL97-precipitated proteins are nuclear proteins, consistent with the fact that pUL97 has been detected mostly, but not exclusively, in a nuclear localization). Importantly, in both cases either using anti-UL97 or anti-cyclin antibodies for CoIP, all known viral substrate proteins of pUL97 could be identified in this measurement, namely pUL44, pUL50, pUL53, pUL69 and pp65 (Table 3, see gene names in bold). In addition, a number of viral proteins were detected in the pUL97 CoIP sample with WSC ratios between 3 and 14, but the functional relevance of this finding is not clear to date. It should be stressed that the mass spectrometry-based detection of short-lived proteins, such as cyclins and highly dynamic cyclin-associated complexes, may be restricted to a low level of peptide counts. The length (amino acids) of all detected proteins is given in Table 1, Table 3 and Table 4, so that this parameter can be set into account of the detected peptide frequencies. In the current experiments, a free range of proteins with lengths between 164 and 2241 amino acids could be detected without any notable size-specific restriction. It should be mentioned, however, that we cannot rule out the possibility of a high false-positive rate. Of note, some of the viral proteins were likewise detected in the cyclin-specific CoIPs, particularly in the cyclin H sample (Table 3, lane H). Here again, the pUL97 substrates were dominantly detected (WSC ratios between 3 and 28), but also for other viral proteins, a putative association with cyclin H was apparent (WSC ratios between 3 and 13). It appears important to mention that this entire group is composed of potential phosphoproteins (i.e., either known phosphoproteins or candidates very likely to be phosphorylated during HCMV replication, such as tegument, polymerase-associated proteins and others [1,60,61]). Therefore, one possible conclusion could be that these proteins collectively represent substrates of phosphorylation mediated by CDK-cyclin or pUL97-cyclin complexes, or both. It should be stressed that as an example of dual phosphorylation by CDKs and pUL97, the viral regulatory protein pUL69 has been identified by our recent studies [13,29,36]. The successful coimmunoprecipitation of almost all relevant CDKs and CDK/cyclin-specific proteins, considered as a parameter of reliability of our setting, was confirmed by the respective portions of the mass spectrometry data (see lower part in Table 3).

### 3.4. Interaction of pUL97 with Cyclin B1, but Not with Other Cyclins, Occurs in a Kinase Activity-Dependent Manner, Suggesting a Cyclin Type-Specific Mode of Interaction

As a particularly interesting feature of our previous studies, the interaction between cyclin B1 and pUL97 has been shown to be strictly dependent on the kinase activity of pUL97. This has been illustrated by the use of pUL97 inhibitors and mutants lacking kinase activity, all leading to a strongly decreased pUL97-cyclin B1 interaction [50]. Bioinformatic modeling supported this mode of interaction and pointed to a possible conformational change within the pUL97 kinase domain that might directly determine its interaction with cyclin B1 [50]. This point has now been underlined by the present data. When treating HCMV-infected cells with the pUL97 inhibitor MBV (10 µM, added one hour before cell harvest at about five days post-infection), pUL97-cyclin B1 was efficiently and selectively inhibited (Figure 2A, lane 2), whereas no or very little impact on cyclin H and cyclin T1 interaction was measured (lanes 4 and 6; note that pUL97-cyclin T1 was close to the detection limit). To confirm the specificity of pUL97-cyclin T1 interaction, an additional experiment under slightly optimized conditions of infection was performed (Figure 2B; four days instead of five days post-infection), again demonstrating the lack of inhibitory effect of MBV towards this interaction, even when pUL97 expression was partially suppressed (lane 3). These findings were then compared to mass spectrometry analysis determined in parallel using equivalent samples. The pUL97-cyclin B1 interaction was likewise detected in this experiment (Table 4, lane B1, WSC 6) and interaction was entirely blocked by MBV treatment (lane B1/MBV, WSC b.c.). This blocking effect of MBV (as confirmed by two independent measurements, data not shown) was found selectively for cyclin B1, since pUL97 interactions with cyclins H and T1 (WSC 11 and 3, respectively) were not substantially changed by MBV treatment (WSC 10 and 4, respectively). Thus, the mass spectrometry analysis strongly underlines the hypothesis that the interaction of pUL97 with cyclin B1 may be restricted to an active state of the pUL97 kinase.

**Figure 3:**
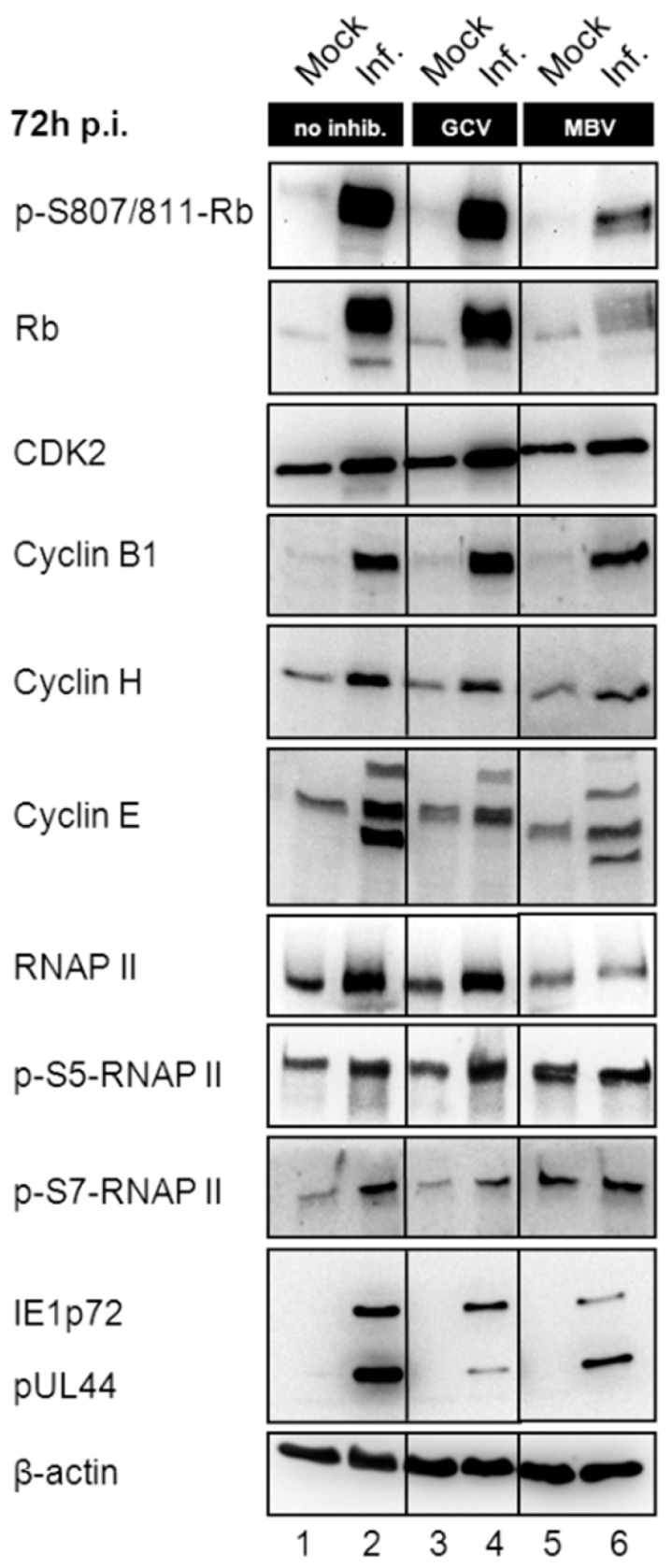
Western blot (Wb) detection of cyclin levels, as well as viral and cellular reference proteins, in mock and HCMV-infected primary fibroblasts, using phospho-specific antibodies. MBV is shown to exert a differential impact on the expression levels of several cellular and viral proteins. HFFs were infected with HCMV AD169-GFP (MOI 0.3) and MBV (30 µM), reference compound ganciclovir (GCV; 20 μM) or DMSO alone (infected; Inf.) was added immediately after infection. Cells were harvested 72 h post-infection (p.i.) and cell lysates were subjected to Wb analysis.

Next, we addressed the question of whether MBV may alter virus-modulated expression of cyclins and other cellular proteins that possibly might be influenced by pUL97 activity. For analyses of protein expression, HFFs remained uninfected or were infected with HCMV AD169-GFP at MOI 0.3 and treated with GCV, MBV or DMSO as a solvent control. At 72 h post-infection, cells were harvested and lysates were subjected to Wb analysis (Figure 3). Expression levels of viral proteins (immediate early and early proteins IE1p72 and pUL44) were monitored and found to be substantially decreased by pUL97-directed inhibitor MBV and GCV when compared to DMSO-treated control cells (Figure 3, lanes 2, 4 and 6; note that this is a refined and extended version of an earlier experiment described in Hutterer et al., 2015; [15]). Then, the expression of various cellular proteins was monitored under conditions of MBV treatment. As a known phenomenon, the tumor suppressor Rb underlies the upregulation and increased phosphorylation of serine residues 807 and 811 during HCMV infection (lane 2). MBV treatment substantially prevented this upregulation and phosphorylation (signal intensities of p-S807/811-Rb quantitated by densitometric evaluation were reduced to 25.0% ± 7.3% by MBV; *n* = 3). In contrast, GCV did not alter the expression level and phosphorylation status of Rb (Figure 3, lane 4). Site-specific phosphorylation of Rb is mediated by pUL97 in 11 of 16 consensus CDK sites leading to its inactivation, thus resulting in the disruption of RbE2F complexes and transcriptional stimulation [27,37]. Surprisingly, the relationship between viral modulation and activity of Rb is more complex than initially supposed, since an experimental knockdown of Rb impaired HCMV replication efficiency at multiple stages [38]. It should be mentioned that in general, Rb is a major repressor of E2F-responsive transcription and restricts cell cycle progression, so that a functional link between Rb expression levels and CDK/cyclin levels in HCMV-infected cells is considered possible. This question was addressed by serial Wb stainings of these samples. CDK2 and cyclins B1, H and E were found upregulated in HCMV-infected cells (Figure 3, lane 2). Importantly, MBV showed no or only a marginal effect on this upregulation (lane 6), thus demonstrating a difference in the effect exerted on Rb. Of particular interest is that cyclin expression levels under MBV treatment were almost similar to DMSO-treated control cells or GCV-treated cells. This finding strongly suggests that modulation of the pUL97-cyclin interaction in the presence of MBV (especially as seen in the loss of cyclin B1 binding) cannot be explained by an overall quantitative reduction of the cyclin pool. In an additional control panel, the previously stated HCMV-conferred activating phosphorylation of RNA polymerase II (RNAP II) on serine residue 5 or 7 of its regulatory C-terminal domain (in part subject to pUL97 activity; [35]) was found partly inhibited by MBV treatment. Thus, the impact of pUL97 activity on levels of expression was detected in a most pronounced fashion for Rb, but was found at marginal levels for RNAP II and CDK2, as well as for cyclins B1, E and H. Overall, the following lines of experimental evidence suggest that the interaction between the cyclin types B1, H and T1 and viral pUL97 may be based on differential modes of binding: Firstly, the strength of the detected interactions varies substantially between the cyclins (cyclin T1 < B1 < H; Figure 1 and Figure 2). Secondly, in samples from transientlypUL97-expressing cells, pUL97-cyclin B1 interaction was mostly detectable, whereas in samples from HCMV-infected cells pUL97 interaction with all three cyclins B1, H and T1 could be detected, as particularly stressed by the data derived from mass spectrometry analyses. Thirdly, the inhibitor sensitivity of pUL97-cyclin association does not appear to be uniform among these complexes, but was specifically found for the pUL97-cyclin B1 interaction.

**Figure 4:**
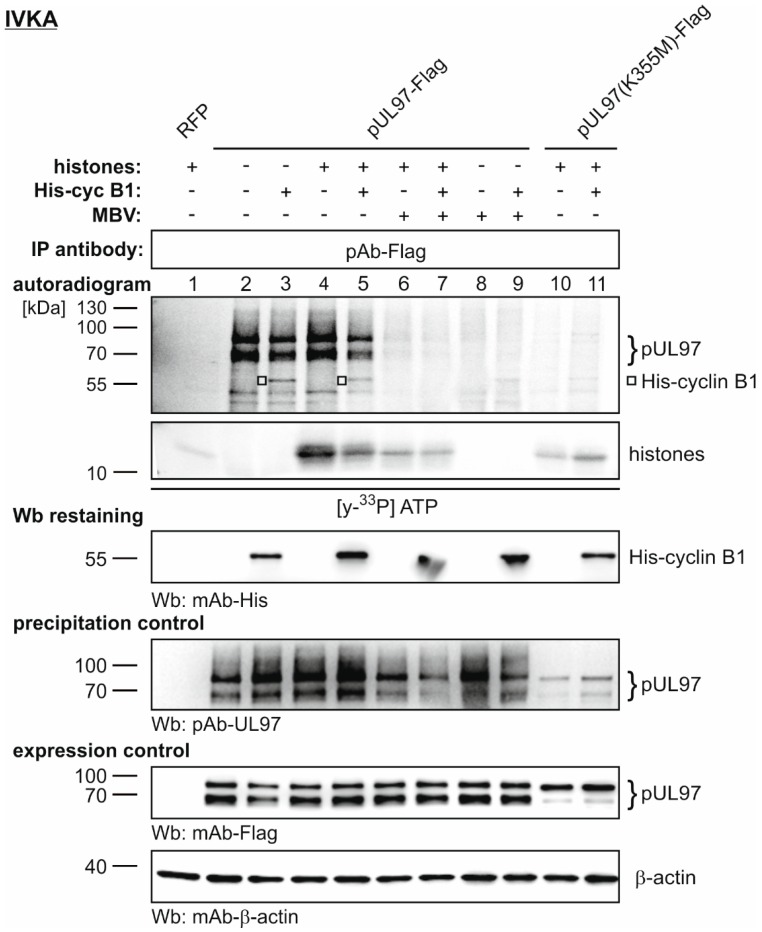
A pUL97-specific in vitro kinase assay (IVKA), determining the putative modulatory effect of cyclin B1 association with pUL97. Transiently expressed pUL97-Flag or pUL97(K355M)-Flag were harvested in a cyclin-depleted fashion by applying high-stringency IP. The two versions of pUL97 were subjected to IVKA reactions under standard conditions. Each reaction was supplemented by the addition of either human cyclin B1 (2 µg), human histones (20 µg) or pUL97 inhibitor MBV (3 µM) as indicated. Upper panels, IVKA (autoradiogram) and detection of His-cyclin B1 on the IVKA membrane (Wb restaining); middle panel, detection of comparable pUL97 levels (precipitation control); lower panels, total input levels contained in cell lysates (expression control); RFP, red fluorescence protein (used as a transfection control).

Finally, we addressed the question whether cyclin association may have a direct impact on pUL97 kinase activity. To this end, we performed a first functional validation on the basis of an in vitro kinase assay. The catalytically active and inactive versions of pUL97, (i.e., pUL97-Flag versus mutant pUL97(K355M)-Flag), were transiently expressed in 293T cells and immunoprecipitated by applying an increased stringency of IP buffer conditions (500 mM of NaCl, in contrast to 150 mM applied in experiments of Figure 1 and Figure 2). This level of IP stringency prevented the cyclin B1 association to pUL97 as demonstrated by IP/Wb analysis (data not shown; notably, the basal pUL97 in vitro activity was not impaired by the high-stringency IP as monitored by control IVKA reactions). Then, a preparation of bacterially produced, purified human cyclin B1 was exogenously added to the pUL97-specific IVKA reactions to investigate putative modulatory effects (Figure 4). Qualitative and semi-quantitative evaluation of the IVKA reactions on the basis of autoradiography/Wb analysis led to the following observations: (i) the basal activity of pUL97 was already present in the cyclin-depleted form (high-stringency IP), i.e., pUL97 autophosphorylation activity was independent from cyclin B1; (ii) the in vitro phosphorylation of cyclin B1 by pUL97 was detectable, thereby suggesting that an active pUL97-cyclin B1 complex was formed by the addition of recombinant cyclin B1; and (iii) the level of histone phosphorylation (here applied as a standard kinase in vitro substrate) was modulated by the addition of cyclin B1, being compatible with the idea of a role of cyclin B1 in pUL97 substrate recognition. The specificity control reactions using MBV and the mutant K355M suggested that no (or very limited) contaminating kinase activity was present in the reactions, i.e., the detected phosphorylation activities were considered pUL97-specific. Note that additional restainings of the IVKA and control blots with antibodies against CDK1 and cyclin B1 confirmed the absence of contaminating CDK1-cyclin B1 activity (data not shown). To our knowledge, this is the first experimental indication arguing for the functional significance of pUL97-cyclin association.

## 4. Discussion

This study highlights the previously identified cyclin association of the HCMV-encoded protein kinase pUL97 and additional viral proteins by providing the first approach using a proteomics-based high resolution analysis accompanied by biochemical and bioinformatic settings. Our data provide novel evidence for the following points: (i) pUL97 undergoes interaction with a subset of, but not all analyzed, human cyclins in HCMV-infected cells (in particular, cyclins H, B1 and T1); (ii) pUL97-cyclin B1 interaction is sensitive to inhibition of pUL97 kinase activity, whereas interaction with cyclins H or T1 is not; (iii) ectopically expressed pUL97 interacts mostly with cyclin B1, in contrast to the multiple cyclin interaction patterns seen with pUL97 expressed in HCMV-infected cells; (iv) cyclin H might be subject to a virus-specific modulation during HCMV replication, since cyclin H was found as the dominant type of cyclin interacting with pUL97 in HCMV-infected cells; and (v) a variety of viral phosphoproteins, amongst them known substrates of pUL97 and CDKs, are found within cyclin-associated complexes.

These findings suggest a new aspect of HCMV-host cell interaction, namely a putative broader regulatory role for cyclin-associated protein complexes during HCMV replication than previously expected, particularly due to the supposed strong impact of cyclin association on pUL97 functionality.

As far as the initial experimental evidence is concerned, pUL97 seems to represent a viral CDK ortholog based on predictions of structural and biochemical similarities with CDKs, primarily CDK2 [26]. The important finding that pUL97 is able to phosphorylate CDK substrates in vitro and in cell-based assays confirms this idea at the functional level [13,26,27,28,35,62,63,64]. The first experimental evidence that pUL97 interacts with human cyclins was provided by Graf et al., (2013); [58], showing that pUL97-cyclin T1 can be coimmunoprecipitated from cotransfected as well as HCMV-infected cells, that interaction can be detected in a yeast two-hybrid assay and that pUL97 colocalizes with cyclin T1 in nuclear compartments. Concerning the selectivity of cyclin binding, it could then be demonstrated that pUL97 interaction with cyclin B1 is much more pronounced than with cyclin T1 and also other types of cyclins, but not all, were detectable associated with pUL97 ([50,58]; data in this report). As a novel finding reported here by employing very sensitive mass spectrometry measurements, additional viral and cellular proteins are suggested to be associated within pUL97-cyclin complexes. Such a situation has already been discussed earlier when exploring dual phosphorylation of the viral early regulator pUL69 by both viral pUL97 and cellular CDKs, primarily CDK9 [13,14,29,36]. This finding led to the hypothesis that cyclin-bridged, higher order protein complexes might combine pUL97- and CDK-mediated phosphorylation of individual substrates. Such multi-protein complexes possibly associating groups of kinases and substrates of the cellular kinome together with viral proteins in HCMV-infected cells, might emphasize the profound role of protein phosphorylation during viral replication. This concept has been substantiated by providing evidence for the high and multifaceted importance of CDK7 and CDK9 in HCMV infection [13,14,15,46,48]. Combined, these threads of experimental evidence argue for a regulatory potential of cyclins, CDKs and herpesviral CDK orthologs that may be more pronounced and diversified in the context of herpesviral replication than previously expected.

**Figure 5:**
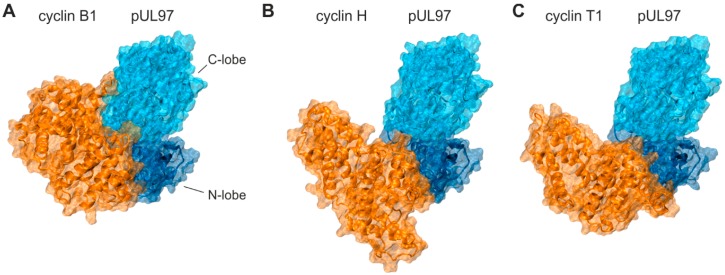
Differences in the interfaces between three types of human cyclins and the kinase domain of viral pUL97 suggested from molecular modeling. Kinase domain of pUL97 with its N-lobe (dark blue) and C-lobe (light blue) in complex with globular regions of: (**A**) cyclin B1; (**B**) cyclin H; and (**C**) cyclin T1. The three cyclins are colored in orange. Note that the globular region of cyclin B1 has contacts to both, the N- and C-lobes of the pUL97 kinase domain, whereas cyclins H and T1 exclusively bind to the N-lobe.

Interestingly, the crystal structures of cellular CDK-cyclin complexes revealed that the mode of interaction may differ significantly depending on the type of cyclin involved [53,63]. Molecular modeling of pUL97 in complex with three types of human cyclins suggests that the differences in the binding interfaces detected in the CDK-cyclin crystal structures [53,54] are also preserved in the respective pUL97-cyclin complexes. The kinase domain of pUL97 can be divided in an amino-terminal lobe (N-lobe) and a larger carboxy-terminal lobe (C-lobe) (Figure 5) with the ATP- as well as inhibitor-binding sites in the cleft between the lobes. The modes of pUL97-cyclin interaction were predicted on the basis of crystal structures of cyclins complexed with human CDKs. For cyclin B1, the suggested cyclin binding interface is rather large and comprises both the N- and the C-lobes of the modeled pUL97 kinase domain (Figure 5A). For cyclins H and T1, different to cyclin B1, the smaller binding interfaces appear to be exclusively restricted to parts of the N-lobe (Figure 5B,C). For this reason, conformational changes within the C-lobe, especially in the activation loop (e.g., mediated through post-translational modification or inhibitor binding), or even changes in the relative orientation of both lobes to each other may poorly influence cyclin H or T1 binding, but may have a strong impact on cyclin B1 binding. In particular, the pUL97 complex with cyclin B1 is rather distinct with respect to the observation that the pUL97 C-lobe is additionally involved in cyclin recognition. This finding is interesting in the light of our previous report [50] indicating that inhibitor binding might affect the conformation of the activation loop, which is located at the intersection between the N- and C-lobes of pUL97. The observations above and the selective effect of MBV on pUL97-cyclin B1 interaction support a model in which binding of MBV might not only affect the conformation of the activation loop but also the interface of cyclin B1 binding, because this cyclin type is specifically interacting with a relatively large surface region of pUL97. Thus, we postulate a cyclin-selective effect of MBV on the basis that inhibition of pUL97 kinase activity may hamper interaction with cyclin B1, but not with cyclins H or T1, by altering structural conformations within the C-lobe. However, considering the fact that the crystal structures of cyclins have not entirely been resolved in all parts, it cannot be excluded that further regions of cyclins may additionally contribute to pUL97 interaction.

From a proteomic point of view, the sensitivity of state-of-the-art nanoliquid chromatography and mass spectrometry couplings offers valuable solutions for investigating interactomes more deeply and, notably, for characterizing viral-cellular multiprotein complexes. On this basis, it is not surprising that a variety of cyclin-associated proteins, mostly of cellular origin, have been identified in coimmunoprecipitates derived from HCMV-infected cell lysates. The question of “true positives” is difficult to address at this stage prior to having collected a larger set of comparative data derived from independent methods. Although we had confirmed the pUL97-cyclin interaction by CoIP, yeast two-hybrid experiments, confocal imaging and now present novel mass spectrometry data, it is currently difficult to categorize the entire list of proteins according to their probability of functional relevance. Considering this factor of uncertainty, it may be important to stress that our study does not claim to provide a complete list of interactors, but rather a group of hits and candidates. We like to stress that on the basis of the current data, conclusions about interactions between viral proteins and cyclins appear reasonable, albeit the capacity of mass spectrometry data is generally limited. Our justification is based on the confirmation of pUL97-cyclin interactions by a number of independent methods for measuring protein-protein interaction. Of note, considering the long list of positives in the mass spectrometry analyses (Tables S1–S3), most of these proteins fall below the cut-off and can be excluded from the final assessment. It will be highly interesting and challenging to validate individual candidates, such as cyclins B1, H and T1, on a functional basis to learn more about their role in co-regulating pUL97 activity. In addition to the findings for pUL97, it was similarly striking to discover a broad level of cyclin association with further viral proteins. This may suggest that larger complexes could be formed, including viral phosphoproteins recruited to protein kinases in a putatively cyclin-bridged manner. The latter aspect has already been discussed in the context of virological and phosphoproteomic analyses focusing on HCMV replication and HCMV-induced modulation of CDK activity [13,39,58,61]. It will, of course, be important to illustrate this scenario in greater detail on a functional level. The coupling of CoIP and quantitative mass spectrometry should allow investigating the timely coordinated changes in the association-dissociation of protein complexes along the course of viral replication. Hence, it is a challenging task to overcome the limits of a static, two-dimensional measurement of protein interactions and to achieve an overview of multiple protein interactions, possibly undergone at different cellular locations at different time points.

The exact regulatory consequences of higher order pUL97-cyclin complexes have remained speculative so far and additional experimentation is necessary to address their general importance, in particular for the outcome of virus replication. The present finding that several types of cyclins are able to interact with pUL97 indicates that cyclin interaction may play an important role not only in the regulation of pUL97 activity and functionality but also in reprogramming host cell activities mainly linked to cell cycle regulation. It has been postulated that pUL97 plays a major regulatory role in HCMV-induced early S phase arrest in HCMV-infected cells, referred to as pseudomitosis [39]. pUL97 obviously contributes to the G1-S phase checkpoint transition through its ability to phosphorylate and inactivate the Rb checkpoint protein [37], but its possible further involvement in cell cycle arrest or other deregulation by putative CDK interaction has not been clarified yet. It is a matter of fact, however, that multimeric complexes consisting of CDKs, cyclins, pUL97 and other viral proteins or substrates are formed during viral replication. Such higher order complexes, as already described by a comprehensive proteomic study by other researchers [65], might be highly dynamic and may possibly be only transiently formed under certain conditions exposing varying kinase activities. In such a scenario, cyclins may play the role of bridging factors triggering phosphorylation events, possibly also including mutual phosphorylation between CDK-cyclin complexes and pUL97. Cyclin binding may mediate or reinforce pUL97 interaction with substrates in a cyclin-dependent fashion. For example, cyclin B1 might mediate phosphorylation of Rb by dual binding of CDK1 and pUL97. Thus, it is an appealing concept that both CDKs and pUL97 might contribute to dual phosphorylation of viral proteins. Whether CDKs compete with pUL97 for the binding of cyclins is still an open question, and thus further data are needed to gain more insight into these regulatory aspects. At present, likely functions of pUL97-cyclin interaction are suggested in the recognition and preference of substrates.

## 5. Conclusions

We conclude from the basis of our findings that cyclin-associated protein complexes, which play important roles in a number of regulatory processes in eukaryotic cells including cell cycle, transcriptional and developmental regulation and signaling, may have a similar importance for HCMV-host cell interaction that might have been underestimated so far. In particular, the viral protein kinase pUL97 has consistently been detected in a cyclin-associated form by various methodological approaches and thus appears to be subject to cyclin regulation. The latter aspect has now been underlined by our findings showing that cyclin B1 interaction is dependent on the active state of pUL97, that the predicted pUL97 binding interfaces for cyclin types are individually distinct (and possibly subject to conformational changes), that cyclin H appears to be modulated during HCMV replication in a way that strongly intensifies pUL97 interaction compared to uninfected pUL97-expressing cells and that higher order pUL97-cyclin complexes can include pUL97 substrates and partly uncharacterized viral phosphoproteins. As deduced from the initial analysis of the pUL97-cyclin B1 interaction on a functional level, our current view favors a role of cyclin binding in pUL97 substrate recognition and preference. Specific experimentation has to be performed in the near future to address the detailed questions of such regulatory potential of cyclin association with pUL97 as well as with other viral proteins. It will be exciting to learn more about this newly recognized example of viral exploitation of the cellular CDK-cyclin proteome.

## Figures and Tables

**Table 1:** CDK/cyclin-specific proteins (selected) including viral protein kinase pUL97 coimmunoprecipitated with antibodies against endogenous cyclins B1, T1 and H using lysates of transiently pUL97-transfected 293T cells *.

Gene	Entry	Length	Protein	WSC [bait]/WSC [Fc-control]			
				B1	T1	H	pUL97
CDK5	Q00535	292	Cyclin-dependent like kinase 5	**5**	-	-	-
CCNB1	P14635	433	Cyclin B1	**34**	-	-	**5**
CDK1	P06493	297	Cyclin-dependent kinase 1	**49**	-	-	**8**
CDK2	P24941	298	Cyclin-dependent kinase 2	**5**	-	-	b.c.
CCNT1	O60563	726	Cyclin T1	-	**49**	-	-
CDK9	P50750	372	Cyclin-dependent kinase 9	**4**	**41**	-	**4**
CCNT2	O60583	730	Cyclin T2	-	**5**	-	-
CCNH	P51946	323	Cyclin H	-	-	**47**	-
CDK7	P50613	346	Cyclin-dependent kinase 7	-	-	**27**	-
MNAT1	P51948	309	Cyclin H assembly factor	-	-	**17**	-
AFF4	Q9UHB7	1163	Major CDK9 EF-associated protein	**3**	**20**	b.c.	**12**
**UL97**	P16788	707	HCMV protein kinase pUL97	**27**	**4**	**5**	**34**

* The 293T cells transiently expressing pUL97-Flag were harvested two days post-transfection for mass spectrometry analyses. Rabbit Fc fragment was used as the reference control for CoIP specificity; b.c., below cut-off (cut-off ≥ 3 and 3-fold enrichment compared to control sample); WSC, weighted spectral counting.

**Table 2:** Quantities of pUL97 interacting with cyclins derived from three different cell systems *.

CoIP Source Material	B1	H	T1	Fc
293T, pUL97 transient transfection	100%	4%	9%	1%
HFF, HCMV AD169 infection **	100%	160%	77%	8%
HFF, pUL97 stable transduction	100%	6%	42%	4%

* Signal intensities were quantitated using Aida Image Analyzer v.4.22 (mean value of three different regions of interest); ** Mean value of four independent experiments harvested at four, five or six days post-infection.

**Table 3:** Viral proteins and CDK/cyclin-specific proteins coimmunoprecipitated with antibodies against endogenous cyclins using lysates of HCMV-infected HFFs *.

Gene	Entry	Length	Protein	WSC [bait]/WSC [Flag-control]								
				A	B1	B2	D1	E	H	T1	pUL97	Fc
**HCMV proteins**												
IRS1	P09715	846	Early protein IRS1	**5**	-	-	-	-	**11**	-	**4**	-
TRS1	P09695	788	Tegument protein HHLF1	-	-	-	-	-	**7**	-	**4**	-
UL104	P16735	697	Portal protein pUL6 homolog	b.c.	-	-	-	b.c.	**3**	-	**7**	-
UL122	P19893	580	IE2	**3**	-	-	-	b.c.	**4**	-	**4**	-
UL24	P16760	358	Tegument protein pUL24	-	-	-	-	-	**5**	-	**6**	-
UL25	P16761	656	Phosphoprotein pp85	b.c.	-	-	b.c.	b.c.	**6**	b.c.	**4**	-
UL26	P16762	222	Tegument protein pUL26	-	-	-	-	b.c.	**8**	-	**6**	-
UL29	C0H677	701	Early protein pUL29/pUL28	-	-	-	-	-	**3**	-	**3**	-
UL32	P08318	1048	Phosphoprotein pp150	b.c.	-	-	-	-	**4**	-	**8**	-
UL35	P16766	640	Tegument protein pUL35	-	-	-	-	-	**5**	-	**7**	-
UL43	P16781	423	Tegument protein pUL43	-	-	-	-	-	**8**	-	**8**	-
**UL44**	P16790	433	DNA polymerase processivity factor pUL44	b.c.	b.c.	b.c.	b.c.	b.c.	**3**	b.c.	**3**	b.c.
UL45	P16782	906	Late protein pUL45	**4**	-	-	-	b.c.	**13**	b.c.	**9**	-
UL47	P16784	983	Capsid assembly protein pUL37 homolog	b.c.	-	-	-	-	**4**	-	**7**	-
UL48	P16785	2241	Deneddylase pUL48	-	-	-	-	-	-	-	**18**	-
**UL50**	P16791	397	Virion egress protein pUL34 homolog	**8**	-	-	-	b.c.	**3**	-	**10**	b.c.
**UL53**	P16794	376	Virion egress protein pUL31 homolog	b.c.	-	-	-	-	b.c.	-	**3**	-
**UL69**	P16749	744	mRNA export factor ICP27 homolog	b.c.	-	-	-	b.c.	**28**	b.c.	**19**	-
**UL83**	P06725	561	Phosphoprotein pp65	b.c.	b.c.	b.c.	b.c.	b.c.	**7**	b.c.	**7**	b.c.
UL84	P16727	586	Early protein pUL84 (viral DNA replication)	-	-	-	-	-	**3**	-	b.c.	-
UL86	P16729	1370	MCP	b.c.	-	-	-	**3**	b.c.	-	**6**	-
**UL97**	P16788	707	Protein kinase pUL97	**5**	b.c.	-	-	**3**	**13**	**3**	**32**	-
US22	P09722	576	Early nuclear protein HWLF1	**22**	-	-	-	b.c.	b.c.	b.c.	-	b.c.
**CDK/cyclin-specific proteins**												
CDK2	P24941	298	CDK2	-	b.c.	-	-	**12**	-	-	-	-
CDK1	P06493	297	CDK1	-	**9**	**6**	-	-	-	-	-	-
CCNB1	P14635	433	Cyclin B1	-	**7**	-	-	-	-	-	-	-
CCNB2	O95067	398	Cyclin B2	-	-	**13**	-	-	-	-	-	-
CCND1	P24385	295	Cyclin D1	-	-	-	**4**	-	-	-	-	-
CCNE1	P24864	410	Cyclin E1	-	-	-	-	**8**	-	-	-	-
CCNH	P51946	323	Cyclin H	-	-	-	-	-	**36**	-	-	-
CDK7	P50613	346	CDK7	-	-	-	-	-	**23**	-	-	-
AFF4	Q9UHB7	1163	Major CDK9 EF-associated protein	-	-	-	-	-	-	**9**	b.c.	-
CCNT1	O60563	726	Cyclin T1	-	-	-	-	-	-	**22**	-	-
CDK9	P50750	372	CDK9	-	-	-	-	-	-	**20**	-	-
MNAT1	P51948	309	Cyclin H assembly factor	-	-	-	-	-	**22**	-	-	-

* HFFs were infected with HCMV AD169 (MOI) and harvested four days post-infection for mass spectrometry analysis. Monoclonal antibody (mAb)-Flag was used as the reference control for CoIP specificity and rabbit Fc fragment served as an additional negative control; b.c., below cut-off (cut-off ≥ 3 and 3-fold enrichment compared to control sample); WSC, weighted spectral counting.

**Table 4:** Viral proteins and CDK/cyclin-specific proteins coimmunoprecipitated with antibodies against endogenous cyclins B1, T1 and H using lysates of HCMV-infected HFFs *.

Gene	Entry	Length	Protein	WSC [bait]/WSC [Flag-control]						
				B1	B1/MBV	H	H/MBV	T1	T1/MBV	Fc
**HCMV proteins**										
IRS1	P09715	846	Early protein IRS1	**3**	**5**	**21**	**15**	-	**6**	b.c.
IR11	P16809	234	Viral Fc-gamma receptor-like protein IR11	b.c.	b.c.	b.c.	b.c.	**4**	**3**	**7**
IRL12	P16810	416	Uncharacterized protein IRL12	b.c.	**3**	-	-	-	-	b.c.
TRS1	P09695	788	Tegument protein HHLF1	**5**	**5**	**12**	**8**	**4**	**3**	b.c.
UL24	P16760	358	Tegument protein pUL24	b.c.	b.c.	**17**	**9**	-	b.c.	-
UL25	P16761	656	Phosphoprotein pp85	b.c.	b.c.	**10**	**9**	b.c.	b.c.	-
UL26	P16762	222	Tegument protein pUL26	**6**	-	**16**	**16**	b.c.	-	-
UL29	C0H677	701	Early protein pUL29/pUL28	b.c.	-	**9**	**9**	-	-	-
UL32	P08318	1048	Phosphoprotein pp150	**21**	**14**	**25**	**27**	b.c.	**6**	-
UL35	P16766	640	Tegument protein pUL35	b.c.	**3**	**10**	**8**	-	-	-
UL43	P16781	423	Tegument protein pUL43	-	-	**11**	**15**	-	-	-
**UL44**	P16790	433	DNA polymerase processivity factor pUL44	b.c.	b.c.	b.c.	**4**	b.c.	b.c.	b.c.
UL45	P16782	906	Late protein pUL45	**11**	**4**	**54**	**51**	**6**	**6**	b.c.
UL46	P16783	290	Triplex capsid protein VP19C homolog	-	b.c.	-	**4**	-	b.c.	-
UL47	P16784	983	Capsid assembly protein pUL37 homolog	-	b.c.	b.c.	**3**	-	b.c.	b.c.
UL48	P16785	2241	Deneddylase pUL48	b.c.	**3**	**11**	**16**	b.c.	**6**	-
**UL50**	P16791	397	Virion egress protein pUL34 homolog	**4**	**3**	**9**	**6**	b.c.	**3**	-
UL52	P16793	668	Packaging protein pUL32 homolog	-	**4**	b.c.	**5**	b.c.	**4**	-
**UL53**	P16794	376	Virion egress protein pUL31 homolog	**3**	**3**	**3**	b.c.	-	b.c.	-
UL54	P08546	1242	DNA polymerase catalytic subunit pUL54	-	-	**3**	b.c.	-	-	-
**UL69**	P16749	744	mRNA export factor ICP27 homolog	b.c.	b.c.	**13**	**16**	b.c.	b.c.	-
UL70	P17149	946	DNA primase pUL70	-	b.c.	**4**	-	-	b.c.	-
UL82	P06726	559	Tegument protein pp71	-	b.c.	**3**	b.c.	-	-	-
**UL83**	P06725	561	Phosphoprotein pp65	b.c.	b.c.	**16**	**15**	b.c.	b.c.	b.c.
UL85	P16728	306	Probable capsid protein VP23	b.c.	**6**	**3**	**9**	b.c.	5	-
UL86	P16729	1370	Major capsid protein MCP	b.c.	b.c.	b.c.	**4**	b.c.	b.c.	b.c.
UL89	P16732	674	Tripartite terminase subunit pUL89	-	b.c.	**4**	**7**	-	**5**	-
UL94	P16800	345	Capsid-binding protein pUL16 homolog	-	**3**	-	b.c.	-	b.c.	-
**UL97**	P16788	707	Protein kinase pUL97	**6**	b.c.	**11**	**10**	**3**	**4**	-
UL98	P16789	584	Alkaline nuclease pUL98	-	**7**	-	b.c.	b.c.	b.c.	-
UL112/UL113	P17151	684	Early phosphoprotein p84	b.c.	b.c.	**4**	**5**	b.c.	b.c.	-
UL122	P19893	580	Immediate early protein 2 (IE2)	b.c.	b.c.	**5**	**6**	b.c.	**3**	-
UL123	P13202	491	Immediate early protein 1 (IE1)	-	**5**	-	b.c.	-	b.c.	-
US22	P09722	576	Early nuclear protein HWLF1	b.c.	**4**	b.c.	b.c.	b.c.	b.c.	b.c.
US23	P09701	592	Tegument protein pUS23	-	-	b.c.	**3**	-	-	-
**CDK/cyclin-specific proteins**											
CCNB1	P14635	433	Cyclin B1	**36**	**29**	-	-	-	-	-
CDKN1B	P46527	198	CDK inhibitor 1B	**7**	**6**	-	-	-	-	-
CDKN1C	B2D1N3	301	CDK inhibitor 1C	**12**	**5**	-	-	-	-	-
CDK1	P06493	297	Cyclin-dependent kinase 1	**42**	**51**	-	-	-	-	-
CDK2	P24941	298	Cyclin-dependent kinase 2	**14**	**15**	-	-	-	-	-
CDK5	Q00535	292	Cyclin-dependent like kinase 5	**5**	**5**	-	-	-	-	-
CDKN1A	P38936	164	CDK inhibitor 1A (Cip1)	**3**	b.c.	-	-	-	-	-
CCNH	P51946	323	Cyclin H	-	-	**33**	**39**	-	-	-
MNAT1	P51948	309	Cyclin H assembly factor	-	-	**15**	**17**	-	-	-
CDK7	P50613	346	Cyclin-dependent kinase 7	-	-	**24**	**23**	-	-	-
AFF4	Q9UHB7	1163	Major CDK9 EF-associated protein	-	-	b.c.	**3**	**12**	**14**	-
CDK9	P50750	372	Cyclin-dependent kinase 9	-	-	-	-	**36**	**39**	-
CCNT1	O60563	726	Cyclin T1	-	-	-	-	**45**	**40**	-

* HFFs were infected with HCMV AD169 (MOI 1) and harvested approximately five days post-infection for mass spectrometry analysis. pUL97 inhibitor MBV (10 µM) was added one hour before harvesting the cells. mAb-Flag was used as the reference control for CoIP specificity and rabbit Fc fragment served as an additional negative control; b.c., below cut-off (cut-off ≥ 3 and 3-fold enrichment compared to control sample); WSC, weighted spectral counting; the filtering of data was performed by the use of Proline software (see Section 2.6) followed by compilation, grouping and comparison of the protein groups as depicted in this Table.

## Supplementary Materials

The following is available online at www.mdpi.com/1999-4915/8/8/219/s1, Table S1: Complete set of mass spectrometry data referring to Table 1, Table S2: Complete set of mass spectrometry data referring to Table 3, Table S3: Complete set of mass spectrometry data referring to Table 4.
